# LPS alters the immuno-phenotype of glioma and glioma stem-like cells and induces in vivo antitumor immunity via TLR4

**DOI:** 10.1186/s13046-017-0552-y

**Published:** 2017-06-22

**Authors:** Sheng Han, Chao Wang, Xiaofei Qin, Junzhe Xia, Anhua Wu

**Affiliations:** Department of Neurosurgery, The First Hospital of China Medical University, Nanjing Street 155, Heping District, Shenyang, 110001 China

**Keywords:** Lipopolysaccharide, glioma stem-like cell, Toll-like receptor 4, Survival

## Abstract

**Background:**

This study examined the ability of lipopolysaccharide (LPS) to affect glioma and glioma stem-like cells (GSCs) in vitro and to induce antitumor immunity in vivo and the role of TLR4 in these processes.

**Methods:**

Using RT-PCR and immunohistochemistry, we examined the expression of TLR4 in 34 glioblastoma clinical samples. Using real time-PCR, western blot and ELISA analyses, the effect of LPS stimulation on the expression of immune related molecules was evaluated in RG2 and U87 GSCs. Control or LPS-pretreated RG2 GSCs were intracranially or subcutaneously implanted into wild-type or nude Fisher 344 rats. Histopathological examinations were used to assess tumor progression and immune infiltration and Kaplan-Meier analyses to compare survival times of the animal models.

**Results:**

TLR4 was highly expressed in glioblastoma clinical samples. In vitro LPS stimulation for 6 h significantly altered expression of immune related molecules in RG2 and U87 GSCs. However, prolonged LPS stimulation diminished this effect. Rats inoculated intracranially with LPS-pretreated RG2 GSCs survived significantly longer than rats inoculated with control RG2 GSCs. In vivo, LPS-pretreated RG2 GSCs expressed higher levels of MHC molecules, CXCL10 and TNF-α and recruited more CD8^+^ lymphocytes. However, intratumoral LPS treatment was not equally beneficial. Furthermore, the in vitro and in vivo effects of LPS stimulation appeared to be largely TLR4-dependent.

**Conclusion:**

LPS pretreatment promotes the recognition and eradication of tumor GSCs in vivo when the immune function of the tumor-bearing host is intact. In addition, our data indicate a complex relationship between bacterial infection and glioma prognosis.

**Electronic supplementary material:**

The online version of this article (doi:10.1186/s13046-017-0552-y) contains supplementary material, which is available to authorized users.

## Background

Glioblastoma is the most malignant glioma that has an extremely poor prognosis [1]. Although surgical resection aims to remove much of the visible tumor mass, it is unable to eradicate invasive and migratory cells [2]. Moreover, glioblastoma cells respond unpredictably to radio-chemotherapy, resulting in further relapses [3]. Glioma stem-like cells (GSCs) that give rise to the heterogeneity of glioma cells also contribute to the resistance to currently available anti-tumor therapies. In addition, the abnormal activation of some signaling pathways augments the stemness and invasiveness of glioblastomas [4]. Due to these treatment obstacles, novel strategies are necessary to improve the outcome of patients with glioblastoma. Immunotherapy is a potential strategy that would allow specific surveillance and eradication of local and distant tumor cells [5].

Glioblastoma creates an immunosuppressive local environment against the body’s immune response, causing immunotherapy failure [5, 6]. Several mechanisms contribute to glioblastoma immune evasion, including down-regulation of MHC and co-stimulatory molecules [7] and up-regulation of immunosuppressive cytokines [8, 9]. Thus, new treatment strategies that employ potent immune stimulation to abrogate local immunosuppression are of particular interest.

Many studies have demonstrated that bacterial infection may affect the prognosis of glioma patients; however, the underlying mechanisms are not yet fully understood. Toll-like receptors (TLRs), which recognize pathogen-associated molecular patterns [10] are expressed and function in both immune cells and tumor cells [11]. Triggering TLRs on immune cells induces pro-inflammatory cytokines, phagocytosis, and immune effector mechanisms. Thus, TLR stimulation is an attractive approach to induce antitumor immunity [12]. Lipopolysaccharide (LPS), a TLR4 ligand and specific bacterial component, has been reported to induce antitumoral effects on glioma cells. However, results from previous studies on TLR4 in glioma remain controversial [12–15]. Therefore, this study sought to further examine the ability of LPS to affect glioma cells and GSCs in vitro and to induce antitumor immunity in vivo.

## Methods

### Cell culture and LPS treatment

Human glioblastoma cell line U87 was purchased from the Chinese Academy of Sciences Cell Bank (Shanghai, China). The rat glioma RG2 cell line was obtained from the American Type Culture Collection (ATCC, Rockville, MD, USA). Cells were grown in Dulbecco’s modified essential media (DMEM, Gibco, Gaithersburg, MD, USA) supplemented with 10% FBS (Hyclone, Logan, UT, USA), 100 U/ml penicillin, and 100 μg/ml streptomycin at 37 °C with 5% CO_2_ in humidified air.

The generation and identification of RG2 and U87 GSCs was performed as we previously described [16]. The characteristic expression of stem markers CD133 and nestin was assessed by immunofluorescence and western blot using antibodies against CD133 (1:100; PA5-38014, Invitrogen, Carlsbad, CA, USA) and nestin (1:250; ab92391 and ab134017, Abcam, Cambridge, UK). The multi-lineage differentiation capacity of GSCs was examined using anti-glial fibrillary acidic protein (GFAP, ab7260; Abcam) and anti-β III tubulin (ab78078; Abcam).

*E. coli* 055:B5 LPS (Sigma, St. Louis, MO, USA) was dispersed in phosphate-buffered saline (PBS) at 1 μg/ml. For in vitro assessments, cells were incubated with LPS for 6, 12, or 24 h and washed three times with PBS before further examination. For in vivo experiments, cells were challenged with LPS for 6 h and washed three times with PBS before animal inoculation.

### Tissue samples

A total of 34 glioblastoma clinical samples were surgically obtained at the Neurosurgery Department of The First Hospital of China Medical University. After resection, samples were immediately snap-frozen in liquid nitrogen. Part of each sample was fixed in formalin, embedded in paraffin wax, and maintained at room temperature for immunohistochemical staining.

### Real time-PCR analyses

Total RNA was extracted using Trizol reagent (Invitrogen) according to the manufacturer’s protocol and 200–500 ng RNA was used for cDNA synthesis. PCR analyses were performed using the primer sets shown in Additional file 1: Table S1. All primers were synthesized by Takara Biotechnology (Dalian, China). Reactions were prepared in triplicate and the conditions were as follows: 95 °C for 3 min, followed by 45 cycles of 95 °C for 20 s, 63 °C for 20 s, and 72 °C for 20 s.

### Western blot analysis

A total cell protein extraction kit (Milipore, Billerica, MA, USA) was used to extract total protein. An equivalent amount of protein from each sample was electrophoresed by 12% SDS-PAGE and transferred to nitrocellulose membrane. After being blocked, membranes were incubated with anti-TLR4 (1:1000; PA5-23124, Invitrogen), anti- MHC-I (1:1000; ab134189 and ab22367, Abcam), anti-MHC-II (1:1000; ab157210 and ab23990, Abcam), anti-CD80 (1:1000; PA5-19211, Invitrogen and bs-2211r, Bioss, Woburn, MA, USA), anti-CD86 (1:1000; bs-1035r, Bioss), anti-TNF-α (1:1000; bs-2081R, Bioss), anti-IL-6 (1:2000; ab9324, Abcam), anti-IL-10 (1:1000; bs-0698r, Bioss), anti-CXCL10 (1:1000; PAA371Ra01; Cloud-Clone Corp., Houston, TX, USA), anti-TGF-β1 (1:1000; c0340, Assay biotechnology, Sunnyvale, CA, USA) and TGF-β2 (1:1000; 5343r-100, BioVision, Milpitas, CA, USA) overnight at 4 °C. Membranes were then washed three times with PBS/0.1%Tween-20 (5 min each), and incubated with a corresponding secondary antibody (1:5000) for 2 h at room temperature. Bands were detected using a chemiluminescence ECL kit (Santa Cruz Biotechnology, Santa Cruz, CA, USA), and were quantified using the Sigma-Gel software (Jandel Scientific Software, Sari Kafael, CA, USA).

### Immunofluorescence

Immunofluorescence stain was performed as described previously [17]. Primary antibodies against MHC- I (1:100; ab22367), MHC-II (1:100; ab23990), CD80 (1:200; bs-2211r), CD86 (1:200; bs-1035r) or CXCL10 (1:100; PAA371Ra01) were used. Cells were observed and imaged with a confocal microscope (Olympus FV1000S-SIM; Olympus, Tokyo, Japan).

### TLR4 gene knockdown

Cells were infected with shRNA lentiviral particles (Santa Cruz Biotechnology) targeting TLR4 (sc-156001-V and sc-40260-V) or control shRNA according to the manufacturer’s protocol and as previously described [17]. Forty-eight hours post-transfection, subcultured cells were selected in 5 μg/ml puromycin for one week. The effectiveness of TLR4 silencing was assessed using western blotting analyses.

### ELISA assays

Cells (1.5 × 10^6^/ml) were cultured for 24 h in serum-containing media in 12-well plates, serum-starved for 4 h, and then stimulated with 1 μg/ml LPS for the indicated times. Cell-free supernatants were collected from treated and control cells. Cytokines were assayed using enzyme-linked immunosorbent assay (ELISA) kits for TNF-α, IL-6 and IL-10 (R&D Systems, Minneapolis, MN, USA) according to the manufacturer’s instructions.

### MTT assays

Cell proliferation was assessed using MTT (3-(4,5-dimethylthiazolyl-2)-2,5-diphenyltetrazolium bromide) assays. Briefly, control or LPS-pretreated cells were seeded in 96-well plates at a density of 2 × 10^3^ cells/well and incubated for 24, 48, 72, 96, or 120 h. At each time point, 20 μl of 5 mg/ml MTT solution was added to each well. After incubation for 4 h, media were removed by aspiration, and formazan crystals were dissolved in 150 μl dimethyl sulfoxide (DMSO). Color intensity was measured at 490 nm using an ELISA plate reader (Tecan Sunrise Remote, Maennedorf, Austria).

### Cell quantification assays

Control or LPS-pretreated cells were seeded in 24-well plates (5 × 10^3^ cells/well) in DMEM supplemented with 10% FBS and grown for 72 h. Cells were then washed by replacing the media with PBS, and trypsinized by adding 200 μl of 0.25% trypsin/EDTA solution. After staining with trypan blue, detached cells were counted using a hemocytometer.

### Transwell assays

Transwell chambers with 8-mm pores (Corning, Corning, NY, USA) were coated with 50 μl Matrigel (BD Biosciences, San Jose, CA, USA). Control or LPS-pretreated cells (2 × 10^3^) were plated in triplicate in 100 μl serum-free DMEM containing 0.1% bovine serum albumin. DMEM with 20% FBS (600 μl) were added to the bottom chamber. Cells were allowed to invade the Matrigel-coated filters for 20 h at 37 °C. Cells that reached the lower surface of the filter were fixed and stained, and counted using a microscope. A total of 10 fields were counted for each filter.

### Neurosphere formation assay

Control or LPS-pretreated GSCs were plated at 200 cells/well in a 24-well plate and grown in the neurosphere culture conditions for 7 days. The neurospheres formed were counted and presented as the percentage of the neurosphere forming cells relative to the 200 cells seeded.

### Preparation and care of animals

Fisher 344 rats and nude rats (weighing 200–250 g) were obtained from the Laboratories Animal Center of China Medical University. All animals were bred under specific pathogen-free conditions at the Laboratories Animal Center of China Medical University.

### Intracranial and subcutaneous implantation of RG2 GSCs in Fisher 344 rats

For intracranial tumor inoculation, rats were deeply anesthetized. A 1-cm midline longitudinal incision was performed, and rats fixed in a rat brain stereotaxic apparatus (Narishige, Tokyo, Japan). A right paramedian craniotomy was made (3 mm lateral and 1 mm anterior to bregma) using a dental drill with a 2-mm bit. Control or LPS-pretreated RG2 GSCs (1 × 10^6^) in 10 μl PBS were stereotactically implanted over 30 s into the right striatum, 5 mm ventral from the cortical surface of the brain. Survival was recorded for each rat from the time of tumor cell implantation.

For subcutaneous tumor inoculation, 1 × 10^6^ control or LPS-pretreated RG2 GSCs in 100 μl of PBS were subcutaneously injected into left flank of wild-type or nude Fisher 344 rats. Tumor growth was monitored by caliper measurement (Precision Instruments Co., Ltd, Shanghai, China) every 2–3 days for 20 days. Tumor volume (V) was calculated as follows: V = L × W^2^ × 0.5, where L is the length and W is the width. Tumor weight was recorded at the end of the study.

### Immunohistochemistry

Paraffin-embedded specimens were cut into 4-μm sections. After deparaffinization with xylene and rehydration, antigen retrieval was performed. Endogenous peroxidase activity was blocked with 3% H_2_O_2_ in methanol. Primary antibodies against TLR4 (1:50), MHC-I (1:100), MHC-II (1:100), CXCL10 (1:100), TNF-α (1:200), or CD8 (1:200; ab33786, Abcam) were used. The immunohistochemical results were evaluated blind [18]. Samples were imaged under a BX-51 light microscope (Olympus Optical Co., Ltd., Tokyo, Japan).

### TUNEL assays

Paraffin-embedded tissues were cut into sections and TUNEL assays were performed using a TdT-FragEL™ DNA Fragmentation Detection Kit (QIA33, Merck) according to the manufacturer’s instructions. Sections were examined and photographed using a light microscope.

### Statistical analyses

Student’s *t*-tests and one-way analyses of variance (ANOVA) were used to assess statistical significance. Differences in survival between the two treatment groups were analyzed using log-rank tests. SPSS 19.0 (SPSS Inc., Chicago, IL, USA) was used for all statistical analyses. Two-tailed *P*-values <0.05 were considered statistically significant.

## Results

### TLR4 is expressed in glioma cells

**Fig. 1:**
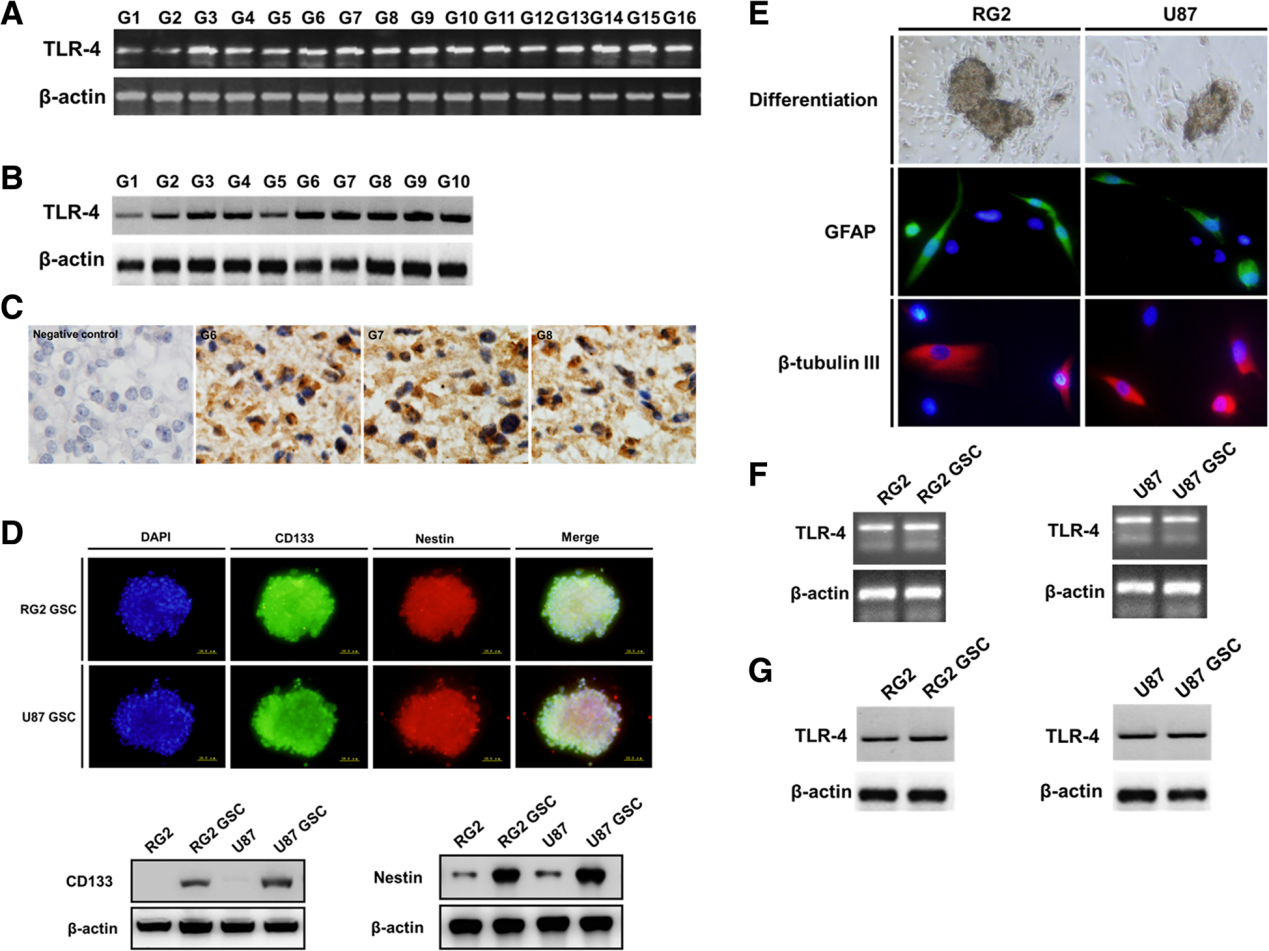
TLR4 expression in glioma cells. Representative images are shown. **a**-**b**: *TLR4* mRNA and protein is expressed in glioblastoma clinical samples as determined by RT-PCR (**a**) and western blot (**b**). **c**: TLR4 protein expression and localization was examined using immunohistochemistry in glioblastoma. **d**: Neurospheres comprised of CD133 and nestin-positive glioma stem-like cells (GSCs) isolated from RG2 and U87 cells. The expression of CD133 and nestin was examined and compared in GSCs and the parental cells by western blot. **e**: GSCs became adherent and differentiated into GFAP- or β III tubulin-positive cells. **f**-**g**: The expression of TLR4 in RG2 and U87 GSCs was determined by RT-PCR (**f**) and western blot (**g**)

All tumor samples tested expressed *TLR4* mRNA and protein (Fig. 1a-b). We used immunohistochemistry to determine the expression and localization of the TLR4 protein in glioblastoma. We confirmed cytoplasmic and membrane localization of TLR4 (Fig. 1c). GSCs were isolated from RG2 and U87 cells, and neural stem cell marker expression and multi-lineage differentiation capacity were examined (Fig. 1d-e). As the markers of cancer stem cells, CD133 and nestin are also associated with the prognosis of glioma patients [19]. Both RG2 and U87 GSCs highly expressed TLR4 (Fig. 1f and g).

### LPS alters the immuno-phenotype of glioma cells and GSCs in a time- and TLR4-dependent manner

**Fig. 2:**
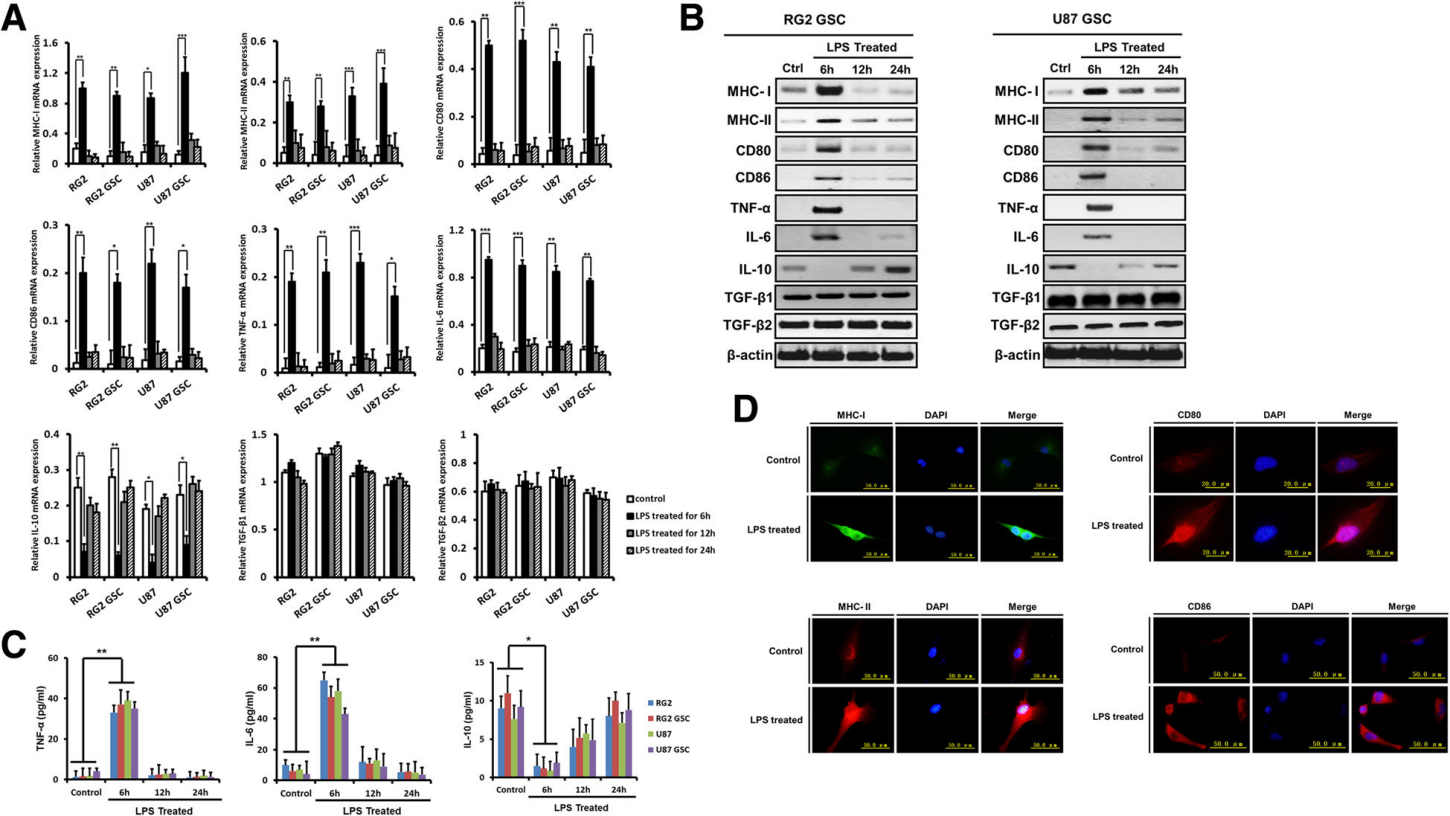
In vitro LPS stimulation alters the immuno-phenotype of glioma cells and GSCs. **a**: After LPS stimulation for 6, 12, or 24 h, the levels of *MHC-I*, *MHC-II*, *CD80*, *CD86*, *TNF-α*, *IL-6*, *IL-10*, *TGF-β1*, and *TGF-β2* mRNA were examined using real time-PCR. **b**: After LPS stimulation for 6, 12, or 24 h, the levels of *MHC-I*, *MHC-II*, *CD80*, *CD86*, *TNF-α*, *IL-6*, *IL-10*, *TGF-β1*, and *TGF-β2* protein in RG2 and U87 GSCs were examined using western blot. **c**: After LPS stimulation for 6, 12, or 24 h, the levels of TNF-α, IL-6, and IL-10 secretion were examined by ELISA in cell-free supernatants from glioma cells and GSCs. **d**: After LPS stimulation for 6 h, the expression of MHC-I, MHC-II, CD80 and CD86 were tested using immunofluorescence stain in RG2 GSCs. Representative images are shown. The results are presented as the mean ± SEM of triplicate samples from three independent experiments. *P <0.05, **P <0.01, ***P <0.001

*MHC-I*, *MHC-II*, *CD80*, and *CD86* were significantly up-regulated, with at least a two-fold increase in their mRNA and protein levels after LPS stimulation for 6 h (Fig. 2a-b). Moreover, real time-PCR, western blot and ELISA assays demonstrated that LPS stimulation for 6 h markedly increased the expression and secretion of the pro-inflammatory cytokines TNF-α and IL-6, but decreased the expression and secretion of the anti-inflammatory cytokine IL-10 (Fig. 2a–c) in RG2 and U87 GSCs. As shown in Fig. 2, the effects of LPS stimulation were most significant at 6 h, and prolonged LPS stimulation decreased these effects. Moreover, TGF-β1 and TGF-β2 expression was not significantly altered with LPS treatment (Fig. 2a-b). Upregulation of MHC-I, MHC-II, CD80, and CD86 was confirmed using immunofluorescence stain in RG2 GSCs after LPS stimulation for 6 h (Fig. 2d).

**Fig. 3:**
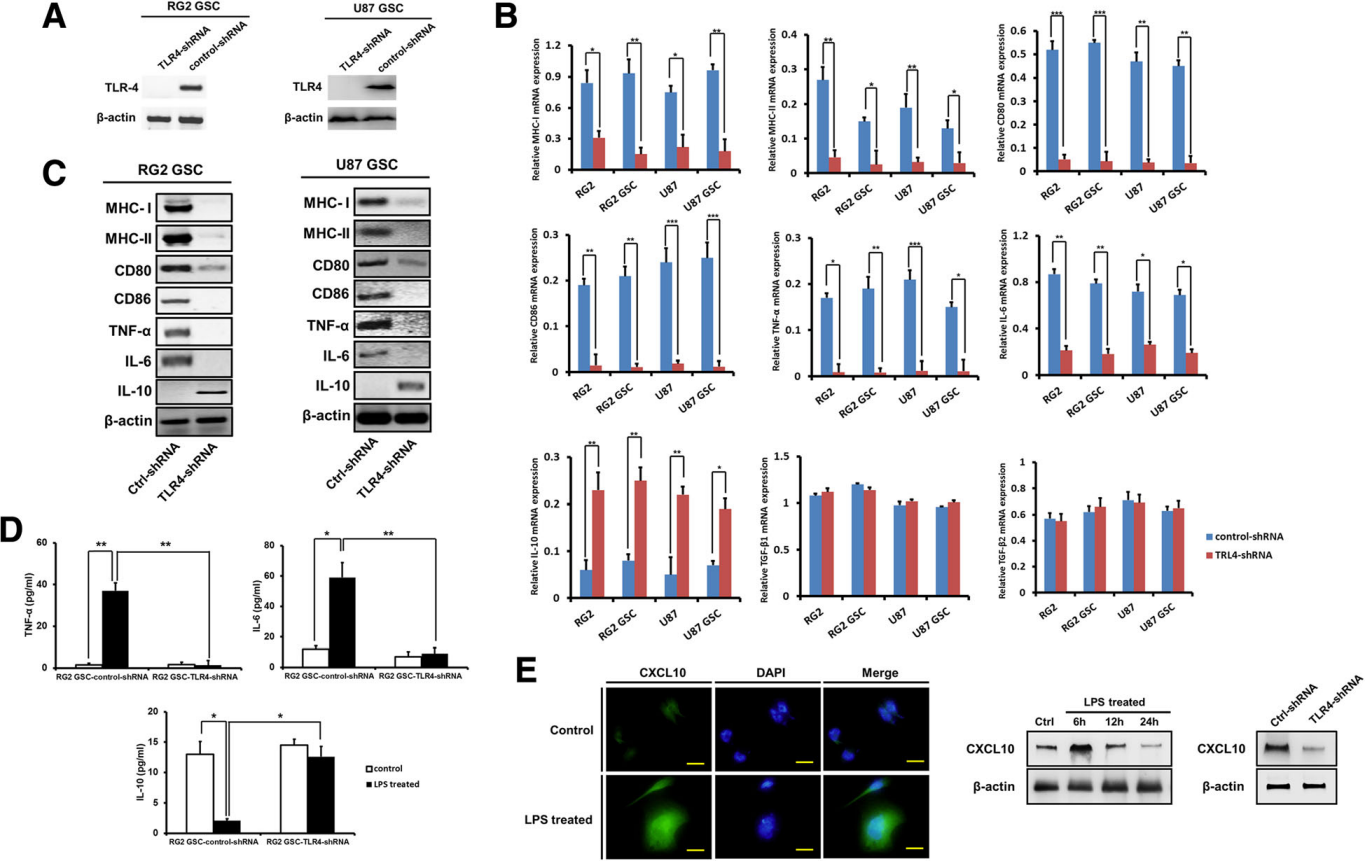
In vitro LPS stimulation alters the immuno-phenotype of glioma cells and GSCs dependent on TLR4. **a**: TLR4 expression was stably knocked down in RG2 and U87 GSCs by specific shRNA as determined by western blot analyses. **b**-**c**: In Control-shRNA cells LPS stimulation for 6 h increased MHC-I, MHC-II, CD80, CD86, TNF-α, and IL-6 expression, but decreased IL-10 expression as determined by real time-PCR (**b**) and western blot (**c**). However, in the presence of *TLR4*-shRNA, the effects of LPS stimulation were abrogated. **d**: After LPS stimulation for 6 h, the levels of TNF-α, IL-6 and IL-10 secretion were detected by ELISA in cell-free supernatants from RG2-control-shRNA and RG2-*TLR4*-shRNA GSCs. **e**: CXCL10 was upregulated in RG2 GSCs after LPS stimulation for 6 h in a TLR4-dependent manner, as determined by immunofluorescence stain and western blot. Scale bars, 25 μm. Representative images are shown. Results are presented as the mean ± SEM of triplicate samples from three independent experiments. *P <0.05, **P <0.01, ***P <0.001

We knocked down *TLR4* in RG2 and U87 GSCs using specific shRNA (Fig. 3A). RG2-control-shRNA cells stimulated with LPS for 6 h increased MHC-I, MHC-II, CD80, and CD86 expression, increased TNF-α and IL-6 expression and secretion, but decreased IL-10 expression and secretion. However, in the presence of *TLR4*-shRNA, the LPS-mediated effects were largely abrogated (Fig. 3). Similar results were obtained in U87-control-shRNA and U87-*TLR4*-shRNA cells. In addition, CXCL10, a chemokine known to recruit CD8^+^ T cells, was also upregulated in RG2 GSCs after LPS stimulation for 6 h in a *TLR4*-dependent manner (Fig. 3e).

Taken together, these data suggest that glioma cells and GSCs are responsive to LPS in vitro. In addition, LPS alters the immuno-phenotype of glioma cells from immunosuppressive to immunoreactive in a time-dependent manner via the TLR4 pathway.

### Short-time LPS stimulation does not -inhibit glioma cell proliferation or invasion

**Fig. 4:**
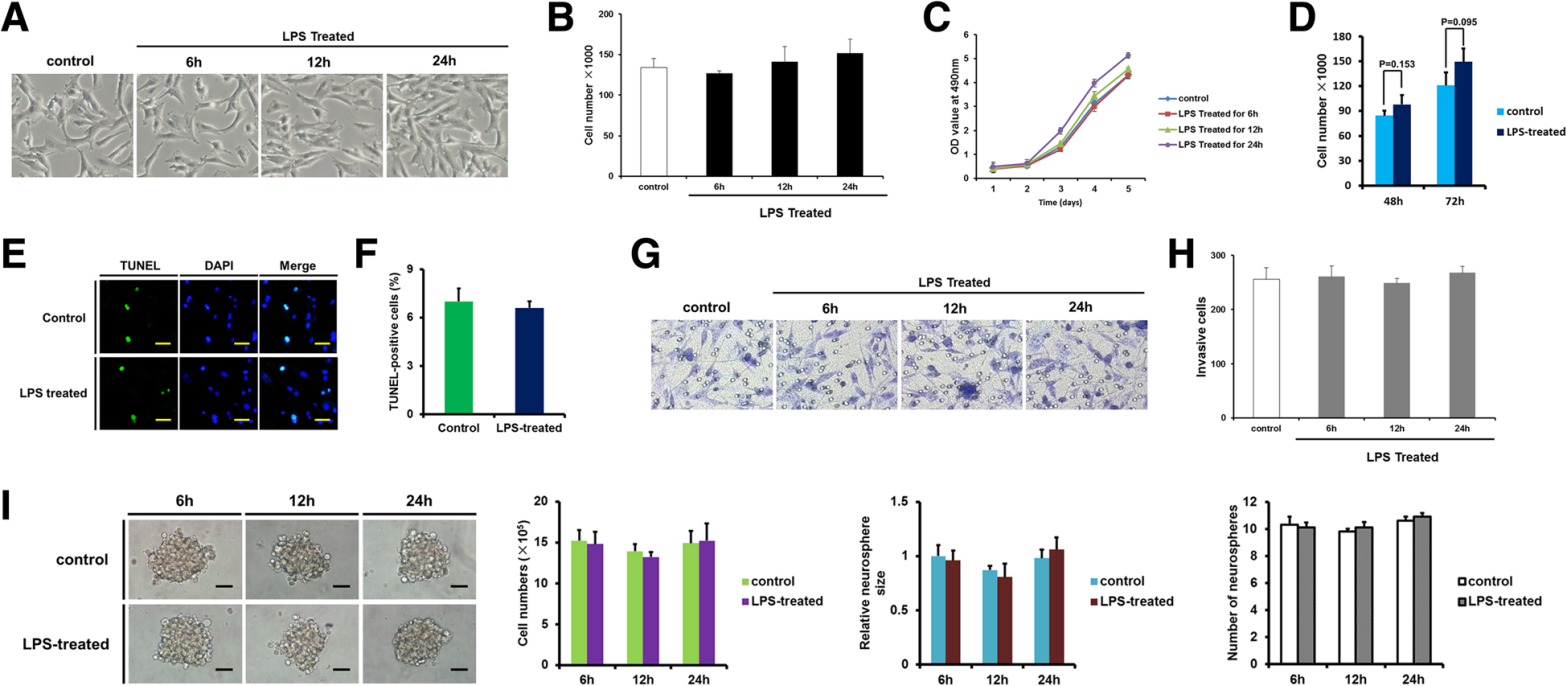
The effect of LPS stimulation on RG2 cell proliferation and invasion. **a**: Representative photomicrographs of control or LPS-treated RG2 cells at different time points. **b**–**c**: The effect of LPS stimulation on cell proliferation was examined by cell counting (**b**) and MTT assays (**c**). **d**: After persistent LPS stimulation for 48 and 72 h, the proliferation of RG2 cells was determined by cell counting. **e**-**f**: After LPS stimulation for 6 h, the apoptosis of RG2 cells was examined by TUNEL assay. **e**: Representative photomicrographs. F: Statistical analyses, the rate of apoptosis was calculated according to the following formula: positive cells/(positive cells + negative cells) × 100%. **g**-**h**: The effect of LPS stimulation for 6, 12, or 24 h on the invasive ability of RG2 cells was determined by Transwell assay. **g**: Representative photomicrographs. **h**: Statistical analyses. I: Self-renewal capacities of RG2 GSCs, as determined by the neurosphere formation assay. Representative images of neurospheres formed by RG2 GSCs and quantitative analyses from three independent experiments are shown. Results are presented as the mean ± SEM of triplicate samples from three independent experiments

MTT, cell counting, Transwell and neurosphere formation assay showed that LPS stimulation for 6 h did not significantly inhibit the proliferation, invasion or self-renewal of RG2 cells and GSCs (Fig. 4a-i). TUNEL assay demonstrated that LPS stimulation for 6 h did not significantly influence RG2 cell apoptosis (Fig. 4e). Consistent with previous results [13, 20], we found that LPS stimulation for more than 24 h tended to increase cell proliferation, although statistically significant results were not obtained in this study (Fig. 4a-d).

### LPS pretreatment inhibits tumor cell intracranial growth via TLR4 in a syngeneic model of glioma

**Fig. 5:**
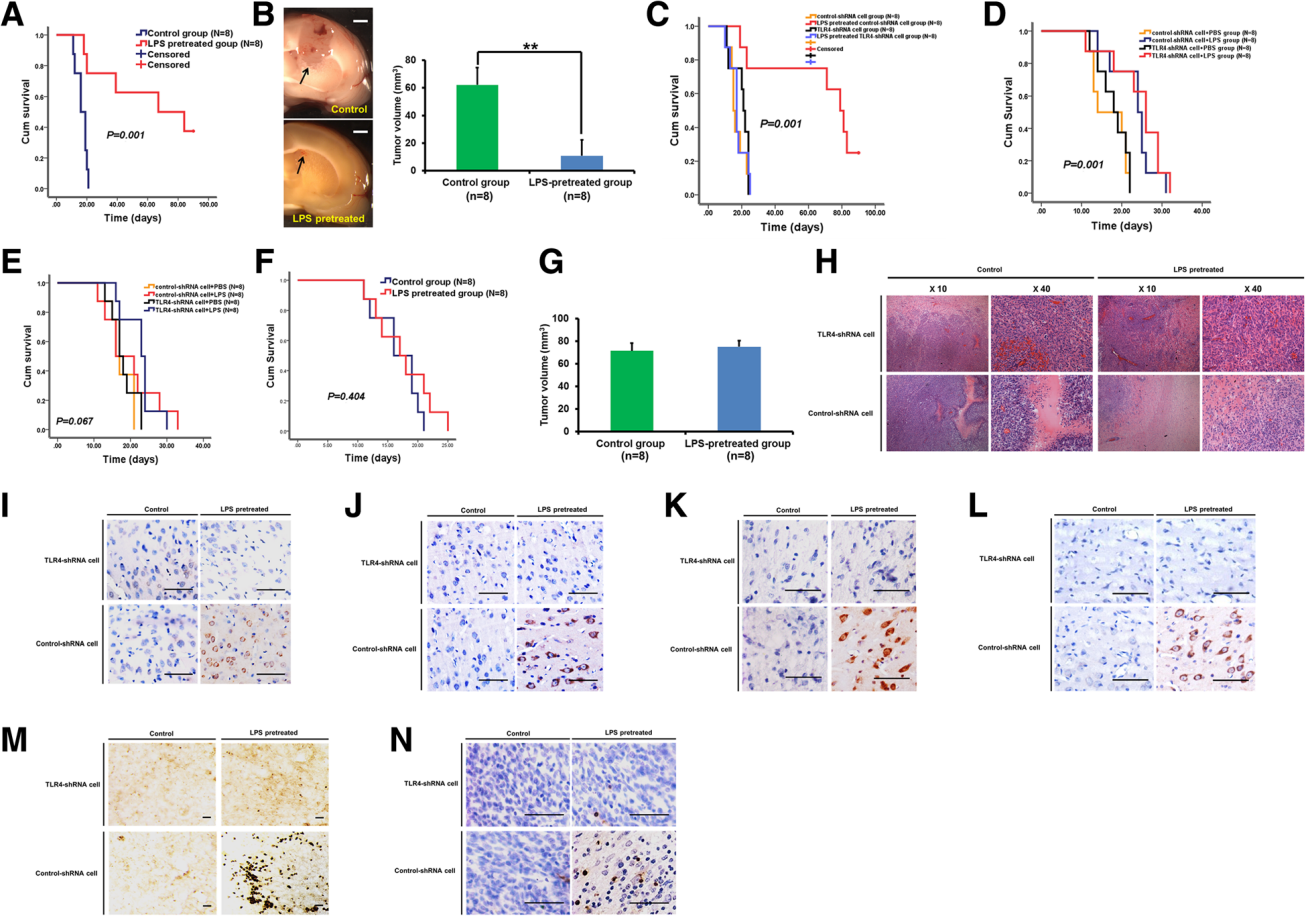
In vivo effects of LPS pre-treatment. **a**: Log-rank analyses of Kaplan-Meier plots showed significantly prolonged survival for wild-type rats inoculated with LPS-pretreated RG2 GSCs. **b**: In wild-type rats with intact immune function, LPS pretreatment significantly inhibited intracranial tumor growth. Left, representative images of intracranial tumor, scale bar 2 mm; right, quantitative analyses. **c**: The survival benefit of LPS pretreatment depends on TLR4 expression in RG2 GSCs. **d**: Log-rank analyses of Kaplan-Meier plots showed a modest increase in survival for LPS-treated (day 0) rats compared with control rats, which was independent of TLR4 expression in RG2 GSCs. **e**: Log-rank analyses of Kaplan-Meier plots showed no significant increase in survival for LPS-treated (day 5) rats compared with control rats. **f**-**g**: For nude rats, the survival (**f**) and intracranial tumor growth (**g**) was not affected in the LPS-pretreated group. **h**-**n**: HE staining (**h**) and immunohistochemical staining shows that LPS pretreatment alters the immuno-phenotype of RG2 GSCs, increases *MHC-I* (**i**), *MHC-II* (**j**), *TNF-α* (**k**), and CXCL10 (**l**) expression, induces CD8^+^ lymphocyte infiltration (**m**), and causes tumor cell apoptosis (**n**, TUNEL staining) in vivo. Scale bars, 50 μm

As shown in Fig. 5a, for wild-type rats, the LPS-pretreated group exhibited significantly longer median survival times (67.0 ± 31.8 days vs. 16.0 ± 2.5 days for the control group), resulting in significantly different survival curves (log-rank tests, *P* <0.001). Tumor growth was determined to be the cause of death for all deceased animals. In the LPS-pretreated group, three rats survived longer than 90 days and exhibited no neurological disabilities. The three rats were protected against a re-challenge with a 2-fold higher dose of untreated RG2 GSCs (2 × 10^6^ cells). In wild-type rats with intact immune function, LPS pretreatment significantly inhibited intracranial tumor growth (tumor volume 10.9 ± 11.4 mm^3^ vs. 62.1 ± 12.6 mm^3^, *P* < 0.001; Fig. 5b). Importantly, the survival advantage of the LPS-pretreated group was abrogated when *TLR4* expression was silenced (Fig. 5c).

Next, we examined the effect of intratumoral LPS treatment. In rats treated on day 0, intratumoral LPS treatment modestly increased survival time (median survival time: LPS-treated 24 ± 2.8 days vs. control 14 ± 5.0 days; *P* <0.001). However, unlike LPS pretreatment, the antitumor effect of intratumoral LPS treatment was independent of TLR4 expression in the implanted glioma cells (Fig. 5d). In rats treated on day 5, intratumoral LPS treatment did not significantly affect tumor growth and survival (*P* = 0.067; Fig. 5e). Moreover, for nude rats with compromised immune function, the survival and tumor growth was not affected in the LPS-pretreated group (Fig. 5f and g).

### LPS pretreatment alters the immuno-phenotype of RG2 GSCs and increases CD8^+^ lymphocyte infiltration in vivo

LPS-pretreated RG2-control-shRNA GSCs expressed higher levels of MHC-I, MHC-II, TNF-α, and CXCL10 in vivo. However, when *TLR4* expression was knocked down, the effects of LPS pretreatment were abolished (Fig. 5h-l). Thus, LPS pretreatment induced an immunoreactive phenotype in RG2 GSCs in vivo that was dependent on TLR4. As shown in Fig. 5m, LPS-pretreated RG2-control-shRNA GSCs attracted significantly more CD8^+^ TILs. In addition, TUNEL staining revealed that the increased number of CD8^+^ TILs correlated with more tumor cell apoptosis in the LPS-pretreated RG2-control-shRNA group (Fig. 5n).

### The immune system of the tumor-bearing host is involved in the antitumor effects of LPS pretreatment

**Fig. 6:**
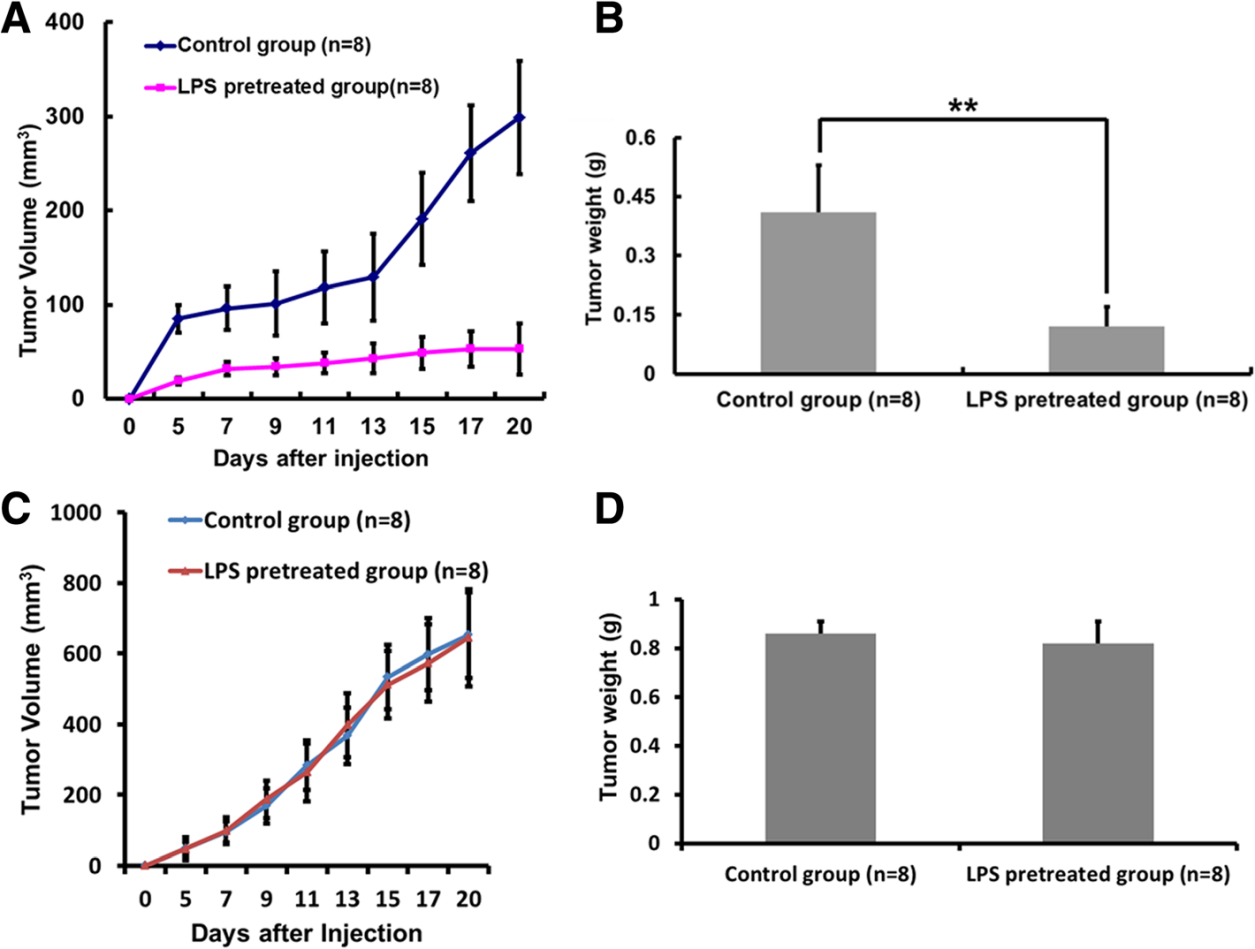
The immune system of the tumor-bearing host is involved in the antitumoral effects of LPS pretreatment. **a** and **b**: In wild-type rats, LPS pretreatment significantly inhibited subcutaneous tumor growth. **c** and **d**: In nude rats, neither tumor growth (**d**) nor the final tumor weight (**d**) were significantly affected by LPS pretreatment. Results are presented as the mean ± SEM. **P <0.01

As shown in Fig. 6a and b, in wild-type rats with intact immune function, LPS pretreatment significantly inhibited subcutaneous tumor growth. However, for nude rats with compromised T-cell function, subcutaneous tumor growth was not affected in the LPS-pretreated group (Fig. 6c and d). These results, together with our in vitro data, demonstrate that short-time LPS pretreatment did not directly inhibit tumor cell proliferation. Therefore, the antitumor effects of LPS pretreatment depend on the host immune system, and the T-cell compartment may be involved in this process.

## Discussion

Glioblastoma is a brain tumor that has a poor prognosis. Despite aggressive surgical resection and concurrent radio-chemotherapy regimens, the prognosis remains dismal with a 2-year survival rate below 27% [1]. Thus, new treatment strategies are urgently needed. Immunotherapy has been intensively investigated as a promising treatment strategy for cancer, and encouraging results have been obtained [8, 18, 21]. However, upregulation of immunosuppressive molecules may restrict T cell response in patients with advanced tumors [22]. Successful immunotherapy relies on stimulation of tumor-reactive immune responses and attenuation of immunosuppression [12].

Because TLRs can modulate both innate and adaptive immunity, TLR ligands are a promising approach for brain tumor immunotherapy [12, 23–25]. To date, most experiments have been designed to activate TLRs on immune system cells, and subsequently activate the antitumor response. However, TLR4 is highly expressed and functional in astrocytes and glioma cells [12, 13, 26] raising questions about the significance of this phenomenon. Moreover, as a well-known TLR4 ligand, LPS has been reported to induce antitumor effects in glioblastoma [15]. Nevertheless, the effects of LPS on glioma were inconsistent, and the underlying mechanism remains unclear [12–15].

In this study, we found that TLR4 mRNA and protein was expressed in glioblastoma clinical samples and glioma cell lines. Furthermore, glioma cells and GSCs are responsive to LPS stimulation via TLR4. LPS stimulation for 6 h resulted in a significant up-regulation of *MHC-I*, *MHC-II*, *CD80*, *CD86*, *CXCL10*, *TNF-α*, and *IL-6* expression but down-regulation of *IL-10*. It has been reported that one potential mechanism of immune paralysis is low expression of MHC-I, MHC-II, CD80, and CD86 in glioma cells, which prevents normal antigen recognition [27–29]. In addition, the tumor microenvironment contains very high levels of tumor-secreted immunosuppressive cytokines, such as IL-10, which contribute to impaired lymphocyte responses in patients with glioma [5, 6]. Indeed, the immunoresistant phenotype of glioma frequently causes the failure of immunotherapy. Depending on TLR4 signaling, LPS stimulation can result in an immunoreactive phenotype of glioma cells and GSCs. Although chronic secretion of pro-inflammatory cytokines can be tumorigenic, it can also promote antitumoral responses depending on the microenvironment [30–32]. Similarly, previous studies have shown that TLR ligands can alter the phenotype of mesenchymal stem cells [33] and malignant B cells [34] rendering them more immunogenic. However, the response of glioma cells to LPS stimulation appears to be time-dependent, and prolonged stimulation diminishes the immuno-activating effect of LPS. Moreover, the effects of LPS stimulation are reported to be long-lasting [35]. Thus, in the present study, RG2 GSCs were washed (after 6 h stimulation) to remove LPS and stop the stimulation before animal inoculation.

Our in vivo experiments showed that depending on TLR4 signaling, LPS pretreatment of tumor cells dramatically prolonged the survival of glioma-bearing rats. This effect was attributed to the altered immuno-phenotype of tumor cells, which caused them to be more immunogenic. Similarly, it has been reported that LPS-stimulated glioma cells can induce a switch in microglial polarization and activation status from an immuno-regulatory phenotype to a cytotoxic and phagocytic phenotype [35] further supporting the immuno-activating effect of LPS stimulation. Surviving rats inoculated with LPS-pretreated RG2 GSCs were protected against a high dose tumor re-challenge, indicating a protective memory response exists in these rats. However, the antitumoral effects of LPS pretreatment were significantly regulated by the tumor-bearing host’s immune system because the inhibitory effects were not observed in nude rats.

The effect of intratumoral LPS treatment remains uncertain. First, bulky, progressively growing tumors at the time of the initial LPS treatment have already created a severe immuno-suppressive microenvironment, which is less responsive to LPS stimulation. As shown previously [12], when intracranial tumors are already established (day 5), LPS treatment is ineffective. In contrast, our findings support previous data [15] showing that on day 0, before obvious tumor growth, LPS treatment significantly increased survival. Additionally, unlike LPS pretreatment, intratumoral LPS treatment may result in long-term stimulation. As shown by our in vitro data, prolonged LPS stimulation compromises its immuno-activating effects. Finally, intratumoral LPS treatment directly affects tumors cells as well as immune cells, thereby explaining why LPS treatment on day 0 has antitumoral effects independent of TLR4 expression in tumor cells. However, the effects of LPS on immune cells of the tumor-bearing host are complex, and some may be detrimental to antitumoral immunity [36, 37]. Intratumoral LPS treatment cannot induce sufficient antitumoral immunity, whereas LPS pretreatment of tumor cells generates an antitumoral milieu at very early stages of tumor implantation and has a superior antitumoral effect. Nevertheless, TLR4 signaling may have different roles in various tissues and cells [11], and the mechanisms should be explored in future studies.

There are isolated reports indicating that patients with glioblastoma who developed infections might live longer [38–40]. It has also been reported that bacterial infection induces antitumoral responses in cancer animal models [41]. Presumably, the infection stimulates the immune responses to attack both pathogens and malignant cells. Our results show that LPS, a specific bacterial component, can modulate the immuno-phenotype of tumor cells, and induce antitumoral effects in certain instances. However, during bacterial infection, LPS might persist in vivo. Our in vitro data suggest that prolonged LPS stimulation not only compromises its immuno-activating effects but also potentially increases glioma cell proliferation. Thus, bacterial infection has uncertain effects on glioma, and the relationship between bacterial infection and cancer outcome is complicated. Bacterial infection cannot be simply used in cancer therapies.

## Conclusion

The bacterial component LPS dramatically alters the immuno-phenotype of glioma cells and GSCs via TLR4 signaling, which enhances glioma immunogenicity and elicits antitumoral immunity, thereby providing a new perspective for glioma immunotherapy. Further studies are required in order to better understand these mechanisms and to discover potential new therapeutic strategies for glioma.

## Additional file

Additional file 1: Table S1. PCR Primers. (DOC 43 kb)
