# Supplementary material for: LPS alters the immuno-phenotype of glioma and glioma stem-like cells and induces in vivo antitumor immunity via TLR4
Source: J Exp Clin Cancer Res. 2017 Jun 22;36:83. doi: 10.1186/s13046-017-0552-y (PMC5480420; doi:10.1186/s13046-017-0552-y)
Supplement: Additional file 1: Table S1. — PCR Primers. (DOC 43 kb) [file 13046_2017_552_MOESM1_ESM.doc]

**Supplementary Table 1. PCR Primers**

| **Primer** | **Forward (5’-3’)** | **Reverse (5’-3’)** |
| --- | --- | --- |
| hTLR4 | TCCTTACCCAGTCCTCATC | CCTCCCTCAAGTCTGTGAA |
| rTLR4 | TTGAAGACAAGGCATGGCATGG | TCTCCCCAAGATCAACCGATG |
| hMHC-I | GGAGGACCAGACCCAGGACA | ACATCACGGCAGCGACCA |
| rMHC-I | TCATTGGAGTTTGTGTGAGGAG | AGTGTGGAAGTGAGGAGCTGAT |
| hMHC-II | GGCTCTTTCGTCACCCTT | CTTCTCCCTGTGCCTTCC |
| rMHC-II | CAAGCGTCAGAAAGGACCTC | TCGAGCTTTGGCAGTATGTG |
| hCD80 | GAAGTGGCAACGCTGTCC | CGTATGTGCCCTCGTCAG |
| rCD80 | AGAGGATTACCTGCTTTGC | CTGCTTGCCTCATTTCTT |
| hCD86 | GGAATGCTGCTGTGCTTA | GGGTCCAACTGTCCGAAT |
| rCD86 | GGGATAACCAGGCTCTAC | ATGTTGTCGCCATACTCA |
| hTNF-α | AGCCCATGTTGTAGCAAACC | AGGCCCCAGTTTGAATTCTT |
| rTNF-α | CCACGCTCTTCTGTCTACTG | GCTACGGGCTTGTCACTC |
| hIL-6 | GGAGACTTGCCTGGTGAA | TGCCCAGTGGACAGGTTT |
| rIL-6 | CCTTCTTGGGACTGATGT | CTCTGGCTTTGTCTTTCT |
| hIL-10 | TTACCTGGAGGAGGTGATGC | TGGGGGTTGAGGTATCAGAG |
| rIL-10 | GCTATGTTGCCTGCTCTT | ATGCTCCTTGATTTCTGG |
| hTGF-β1 | GCTGCTGTGGCTACTGGTGC | ACCTCCTTGGCGTAGTAGTCG |
| rTGF-β1 | CCGCAACAACGCAATCTA | TGAGGAGCAGGAAGGGTC |
| hTGF-β2 | TTGACGTCTCAGCAATGGAG | TGCAGCAGGGACAGTGTAAG |
| rTGF-β2 | ATCCCGCCCACTTTCTAC | CCGTTGTTCAGCCACTCT |
| hβ-actin | CTCCATCCTGGCCTCGCTGT | GCTGTCACCTTCACCGTTCC |
| rβ-actin | GAGAGGGAAATCGTGCGTGAC | CATCTG CTGGAAGGTGGACA |

h: homo sapiens; r: rattus norvegic
